# Papez circuit change following ventriculoperitoneal shunt for hydrocephalus: a case report

**DOI:** 10.1007/s13760-021-01661-x

**Published:** 2021-04-10

**Authors:** Min Kyeong Cho, Sung Ho Jang

**Affiliations:** Department of Physical Medicine and Rehabilitation, College of Medicine, Yeungnam University, 317-1, Daemyungdong, Namku, Taegu, 705-717 Republic of Korea

**Keywords:** Papez circuit, Cognitive impairment, Diffusion tensor imaging, Hydrocephalus, Ventriculoperitoneal shunt

Dear Editor,

Hydrocephalus is a dilatation of the brain ventricular system and is usually accompanied by an elevated intraventricular pressure [1]. The increased pressure associated with the hydrocephalus can compress brain white matter adjacent to ventricles, and various clinical presentations may appear in the chronic hydrocephalus in adult results depending on the age of the patient, the co-existence of cerebrovascular disease, and the presence or absence of intracranial hypertension [1, 2]. Successful shunt operation for hydrocephalus management can result in decompression of the compressed white matter or neural tracts around the ventricles [1]. (Supplementary file 1).

The Papez circuit, initially described by James Papez, is involved in memory function, particularly episodic memory. The Papez circuit is comprised of various brain structures and neural tracts, and the circuit consists of the hippocampus—fornix—mammillary body—anterior thalamic nucleus—cingulate gyrus—cingulum—parahippocampal gyrus—hippocampus [3]. Because of the anatomical characteristics of the Papez circuit (deep location within the subcortical white matter and difficult discrimination from adjacent neural structures), accurate estimation of the Papez circuit on conventional brain computed tomography (CT) or magnetic resonance imaging (MRI) has been limited in the live human brain. However, the recent development of diffusion tensor tractography (DTT), a tract imaging system derived from diffusion tensor imaging (DTI) data, enables estimation of the Papez circuit via three-dimensional reconstruction of neural tracts [4].

An advantage that is unique to DTT is its capacity to determine changes in neural tracts, such as those of the Papez circuit, by performing configurational analysis and three-dimensional reconstruction of the tracts of interest [4]. Furthermore, the state of the Papez circuit can be determined by assessing changes in various DTT parameters, such as the fractional anisotropy (FA) and tract volume (TV) parameters. The FA value represents the state of white matter organization as it indicates the degree of directionality and integrity of white matter microstructures, whereas the TV value represents the number of voxels included in a neural tract, thereby suggesting the total number of fibers within that tract [5] (Supplementary file 1).

In the current study, we report on a stroke patient who showed changes in the Papez circuit on follow-up DTTs and a reduction in cognitive impairment following ventriculoperitoneal (VP) shunt for hydrocephalus.

The fifty-year-old male patient was diagnosed with spontaneous intracerebral hemorrhage in the right frontal lobe, as well as subarachnoid hemorrhage and intraventricular hemorrhage. The patient underwent coiling for a ruptured anterior communicating artery aneurysm, stereotactic drainage, and decompressive craniectomy at the neurosurgery department of a university hospital. Subsequently, he was additionally diagnosed with hydrocephalus and underwent VP shunt placement at the neurosurgery department of the same university hospital three months after stroke onset (Fig. 1a). After VP shunt placement, his cognitive score improved by 11 points on Mini-Mental State Examination (full score: 30, cutoff score < 25; Mini-Mental State Examination score of 6 [2 days before VP shunt] increasing to 17 [6 days after VP shunt]). The patient provided informed consent, and the study protocol was approved by our institutional review board.

**Fig. 1:**
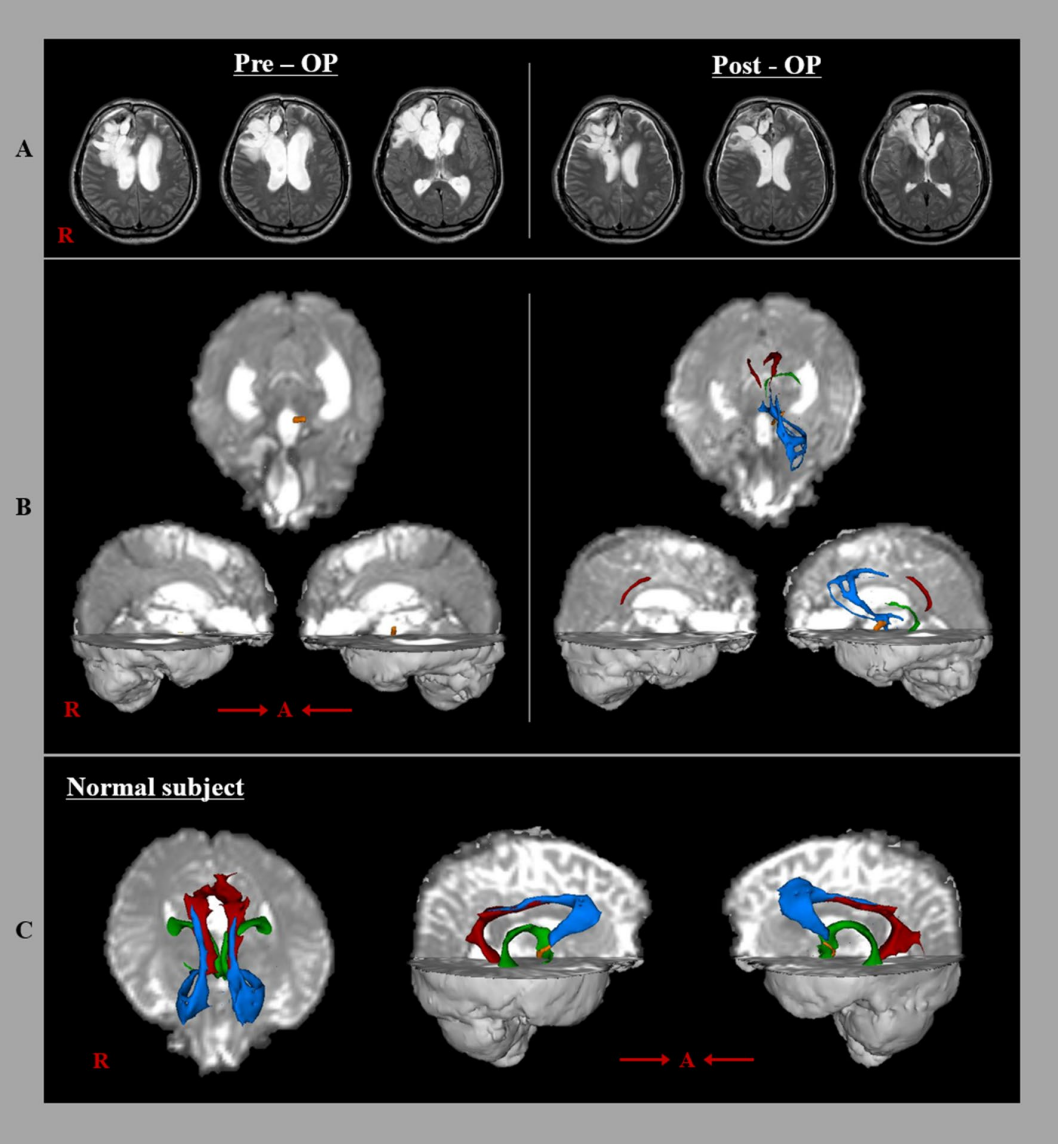
**a** The pre-operative T2-weighted brain magnetic resonance images show dilatation of the ventricular system, as well as improvement of dilatation of the ventricular system, after ventriculoperitoneal (VP) shunt installation. **b** Results of diffusion tensor tractography (DTT) for each neural tract of the Papez circuit of the patient: The left mamillothalamic tract (yellow) is reconstructed, whereas other neural tracts of the Papez tract are not reconstructed on pre-VP shunt DTT. By contrast, the left mamillothalamic tract is thickened, and the left thalamocingulate tract (sky-blue), fornix (green), and both cinguli (red) are reconstructed on post-VP shunt DTT. **c** Results of DTT for each neural tract of the Papez circuit of a normal subject (35-year-old male): thalamocingulate tract (sky-blue), fornix (green), cingulum (red), and mamillothalamic tract (yellow) (color figure online)

DTI was performed twice, two days before and six days after VP shunt placement. On pre-VP shunt DTT, the left mammillothalamic tract (MTT) was successfully reconstructed, whereas other neural tracts of the Papez tract were not reconstructed. By contrast, the left MTT was observed to have thickened, and the left thalamocingulate tract and fornix, and both cinguli were reconstructed on post-VP shunt DTT (Fig. 1b). On post-VP shunt DTT, the FA and TV values for the MTT tract in the left hemisphere were higher than on pre-VP shunt DTT (FA: 0.24 [pre-VP shunt DTT] → 0.42 [post-VP shunt DTT]; TV: 97 [pre-VP shunt DTT] → 125 [post-VP shunt DTT]). The reconstruction of the thalamocingulate tract, fornix, cingulum, and MTT indicates the restoration of these compressed neural tracts following the relief of high intraventricular pressure of hydrocephalus via the VP shunt. The increased FA and TV value of the left MTT after VP shunt indicate increased directionality and number of neural fibers of these neural tracts, respectively [5]. The DTT-observed restoration of the Papez circuit appears to coincide with an improvement in the patient’s Mini-Mental State Examination score, which indicates that the patient’s cognitive impairment has been reduced. Based on these results, we suggest that the use of a VP shunt for hydrocephalus management induced a relatively rapid restoration of the Papez circuit in this patient. (Supplementary file 1).

To the best of our knowledge, this is the first study to use DTT to demonstrate changes in the Papez circuit following VP shunt placement for hydrocephalus in an adult patient who also showed improvement in cognition level. Further complementary studies, including larger numbers of patients, are warranted. In conclusion, by using follow-up DTTs, we demonstrated changes in the Papez circuit in a patient with hydrocephalus who also showed a reduction of cognitive impairment following VP shunt placement. Our results suggest that a VP shunt can affect the state of the Papez circuit in patients with cognitive impairment due to hydrocephalus.

## Supplementary Information

Below is the link to the electronic supplementary material.

Supplementary file1 (PDF 295 KB)
