# Supplementary material for: Papez circuit change following ventriculoperitoneal shunt for hydrocephalus: a case report
Source: Acta Neurol Belg. 2021 Apr 10;122(2):547–9. doi: 10.1007/s13760-021-01661-x (PMC8986659; doi:10.1007/s13760-021-01661-x)
Supplement: Supplementary file 1 — Supplementary file1 (PDF 295 KB) [file 13760_2021_1661_MOESM1_ESM.pdf]

SUPPLEMENTAL MATERIAL (Acta Neurologica Belgica)

Title: Papez circuit change following ventriculoperitoneal shunt for hydrocephalus: A case report

Authors

Author 1: Min Kyeong Cho, Bs

Department of Physical Medicine and Rehabilitation, College of Medicine, Yeungnam University

Mailing Address: Department of Physical Medicine and Rehabilitation, College of Medicine, Yeungnam University 317-1, Daemyungdong, Namku, Taegu, 705-717, Republic of Korea

Tel: 82-53-620-4098 FAX: 82-53-625-3508 e-mail: jmk09609@daum.net

Author 2(corresponding author): Sung Ho Jang, MD

Department of Physical Medicine and Rehabilitation, College of Medicine, Yeungnam University

Mailing Address: Department of Physical Medicine and Rehabilitation, College of Medicine, Yeungnam University 317-1, Daemyungdong, Namku, Taegu, 705-717, Republic of Korea

e-mail: strokerehab@hanmail.net

A ventriculoperitoneal(VP) shunt is the most widely used application for the management of hydrocephalus as such a shunt can divert cerebrospinal fluid into the peritoneal cavity through a catheter[1,2]. Previous studies have reported on the reduction of cognitive impairment after VP shunt placement for hydrocephalus, and the mechanisms associated with the improvement in cognition following VP shunt have been mainly ascribed to decompression of the pressure on the periventricular white matter following VP shunt placement[3,4]. Several studies have reported that Papez circuit injury is related to cognitive impairment[5-7]. Several studies have used diffusion tensor tractography(DTT) to describe changes in white matter or in several neural tracts, such as the cingulum and corticoreticular pathway, following VP shunt placement for hydrocephalus management[3,4,8,9]. By contrast, no study on changes to the Papez circuit following VP shunt placement for hydrocephalus has been reported.

diffusion tensor imaging(DTI) was performed twice, two days before and six days after VP shunt placement, on a 1.5 T Philips Gyroscan Intera (Philips, Ltd., Best, Netherlands) with a 6-channel head coil and using single-shot echo-planar imaging. For each of the 32 non-collinear diffusion-sensitizing gradients, we acquired contiguous slices parallel to the anterior commissure–posterior commissure line. Imaging parameters were as follows: acquisition matrix = 96 × 96; reconstructed to matrix = 192 × 192 matrix; field of view = 240 mm × 240 mm; TR = 10,398 ms; TE = 72 ms; parallel imaging reduction factor (SENSE factor) = 2; EPI factor = 59; b = 1000 s/mm^2; NEX = 1; and slice thickness = 2.5 mm.

Diffusion-weighted imaging data were analyzed by using tools within the Oxford Centre for Functional Magnetic Resonance Imaging of the Brain (FMRIB) Software Library (FSL; www.fmrib.ox.ac.uk/fsl). Affine multi-scale two-dimensional registration was used for correction of head motion effects and image distortion due to eddy currents. Fiber tracking was performed using a probabilistic tractography method based on a multifiber model and was applied in the present study by utilizing tractography routines implemented in FMRIB Diffusion software (5000 streamline samples, 0.5 mm step lengths, curvature thresholds = 0.2; corresponding to a minimum angle of 80°)[10]. Each neural tract within the Papez circuit was determined by selecting fibers passing through seed and target regions of interest(ROI) as follows: thalamocingulate tract—the cingulate gyrus on the axial image(seed ROI), anterior limb of the internal capsule on the axial image(target ROI-1) and the anterior thalamic nuclei on the coronal image(target ROI-2); fornix—the mammillary body on the axial image(seed ROI) and the crus of the fornix(target ROI); mammillothalamic tract(MTT)—the anterior thalamic

nuclei on the axial image(seed ROI), the isolated MTT(target ROI-1), and the mammillary body on the axial image(target ROI-2); cingulum—the middle(seed ROI) and posterior(target ROI-1) of the cingulum on the coronal images and the hippocampal cortex on the axial image (target ROI-2)[11-13]. Out of 5000 samples generated from the seed voxel, contact results were visualized for analysis at a threshold of a minimum of two streamlines through each voxel. The FA and TV values of the patient's DTT-reconstructed Papez circuit were obtained.

Since the introduction of DTT, several studies have reported changes in white matter or neural tracts in adult patients with hydrocephalus after stroke that were treated with a VP shunt[3,4,8,9]. In 2016, Lee and Jang reported on a patient with hydrocephalus after intracerebral and intraventricular hemorrhages who showed reconnection of the compressed left anterior cingulum to the basal forebrain and a reduction in cognitive impairment following VP shunt placement[3]. In 2017, Jang et al. demonstrated increased the neural connectivity of the caudate nucleus to the medial prefrontal cortex on both hemispheres in a patient with hydrocephalus following an aneurysmal subarachnoid hemorrhage who also showed recovery of akinetic mutism after VP shunt placement[4]. Subsequently, Jang et al. [2018] reported on a patient with hydrocephalus after intraventricular hemorrhage who presented restoration of the discontinued corticoreticular pathways in both hemispheres and reduction of gait disturbance following VP shunt placement[8]. Recently, Jang and Lee [2019] reported increased the neural connectivity of the thalamic intralaminar nuclei to the prefrontal cortex in both hemispheres after VP shunt placement in a patient with hydrocephalus after subarachnoid and intraventricular hemorrhages who also showed restored consciousness[9]. Restoration of neural tracts by decompression of the ventricles after VP shunt placement that has been reported in previous studies appears consistent with the results of the present case study.

References

1 Bondurant CP, Jimenez DF (1995) Epidemiology of cerebrospinal fluid shunting. Pediatr Neurosurg 23:254-258; discussion 259.

2 Reddy GK, Bollam P, Shi R, Guthikonda B, Nanda A (2011) Management of adult hydrocephalus with ventriculoperitoneal shunts: long-term single-institution experience. Neurosurgery 69:774-780; discussion 780-781.

3 Lee H, Jang SH (2016) Change of cingulum following shunt operation for hydrocephalus in a patient with a haemorrhagic stroke. Clin Neurol Neurosurg 148:49-51.

4 Jang SH, Chang CH, Jung YJ, Lee HD (2017) Recovery of akinetic mutism and injured prefronto-caudate tract following shunt operation for hydrocephalus and rehabilitation: a case report. Medicine (Baltimore) 96:e9117.

5 Chang MC, Yeo SS, Do Lee H, Jang SH (2016) Diffusion tensor tractography in a patient with memory impairment following encephalitis. Acta Neurol Belg 116:629-631.

6 Jang SH, Kwon HG (2016) Neural injury of the Papez circuit following hypoxic-ischemic brain injury: a case report. Medicine (Baltimore) 95:e5173.

7 Jang SH, Kwon HG (2018) Injury of the Papez circuit in a patient with traumatic spinal cord injury and concomitant mild traumatic brain injury. Neural Regen Res 13:161-162.

8 Jang SH, Chang CH, Jung YJ, Seo YS (2018) Restoration of the corticoreticular pathway following shunt operation for hydrocephalus in a stroke patient. Medicine (Baltimore) 97:e9512.

9 Jang S, Lee H (2019) Change of ascending reticular activating system following shunt operation for hydrocephalus in a subarachnoid hemorrhage patient. J Neurol Surg A Cent Eur Neurosurg 80:62-66.

10 Smith SM, Jenkinson M, Woolrich MW, et al (2004) Advances in functional and structural MR image analysis and implementation as FSL. Neuroimage 23 Suppl 1:S208-219.

11 Concha L, Gross DW, Beaulieu C (2005) Diffusion tensor tractography of the limbic system. AJNR Am J Neuroradiol 26:2267-2274.

12 Kwon HG, Hong JH, Jang SH (2010) Mammillothalamic tract in human brain: diffusion tensor tractography study. Neuroscience Letters 481:51-53.

13 Jang SH, Yeo SS (2013) Thalamocortical tract between anterior thalamic nuclei and

cingulate gyrus in the human brain: diffusion tensor tractography study. Brain Imaging Behav 7:236-241.
